# Patient Perceptions of Ozempic (Semaglutide) for Weight Loss: Mixed Methods Analysis of Online Medication Reviews

**DOI:** 10.2196/78391

**Published:** 2026-01-09

**Authors:** Abanoub J Armanious, Rachel-Mae Hunter, Kristi R Griffiths, Hannah E Bowrey, Robyn M Brown, Morgan H James

**Affiliations:** 1 Department of Psychiatry Rutgers Robert Wood Johnson Medical School Piscataway, NJ United States; 2 Rutgers Addiction Research Center, Brain Health Institute Rutgers, The State University of New Jersey Piscataway, NJ United States; 3 InsideOut Institute Faculty of Medicine and Health The University of Sydney and Sydney Local Health District Sydney Australia; 4 School of Psychology, Faculty of Science The University of Sydney Camperdown Australia; 5 Brain and Mind Centre The University of Sydney Camperdown Australia; 6 Department of Biochemistry and Pharmacology The University of Melbourne Parkville Australia

**Keywords:** Thematic analysis, discussion board, pharmacotherapy, glucagon like peptide 1, GLP-1, incretin mimetic, craving.

## Abstract

**Background:**

Ozempic (semaglutide) has received widespread attention for its appetite-suppressing effects, leading to extensive off-label use for weight loss. Although gastrointestinal side effects are well documented, less is known about how users assess the trade-off between perceived benefits and adverse effects, or how these assessments influence treatment discontinuation. Importantly, existing insights are often limited to clinical trial populations and may not fully reflect real-world experiences.

**Objective:**

This study applies a novel infoveillance approach to examine patient-reported experiences with off-label Ozempic use for weight loss and to identify the factors most strongly associated with user satisfaction and treatment discontinuation.

**Methods:**

We analyzed 60 publicly available, self-selected, anonymous user reviews of Ozempic from Drugs.com. Reviews were initially examined using thematic analysis to identify key themes describing patients’ lived experiences with treatment. These qualitative themes were then linked to user-provided ratings of perceived drug efficacy (1-10 scale) and statements regarding intent to continue or discontinue treatment. This mixed methods approach enabled the integration of qualitative depth with quantitative patterns within naturally occurring, deidentified online data.

**Results:**

Three major themes emerged from the thematic analysis: (1) change in body weight and appetite, (2) nonweight-related symptoms and side effects, and (3) plans for ongoing use versus discontinuation. Two-thirds of respondents reported reduced appetite, food cravings, or body weight. Gastrointestinal complaints were common (reported by 37 of 60, 62%, reviewers) but did not significantly (*P*=.39) influence satisfaction ratings or decisions to continue treatment. Instead, minimal/no weight loss and the emergence of nongastrointestinal side effects were more frequently associated with low overall satisfaction and discontinuation. Effective weight loss, even when accompanied by gastrointestinal side effects, was associated with a greater willingness to continue Ozempic treatment.

**Conclusions:**

This study presents a novel application of infoveillance methods to characterize real-world patient attitudes toward off-label Ozempic use. Satisfaction was driven primarily by perceived effectiveness rather than tolerability. Key limitations are the self-selected nature of the sample, reliance on anonymous, self-reported data, and the lack of demographic, dosing, or treatment-duration information. Nonetheless, these findings underscore the value of online health forums as a rich and underutilized source of patient-centered insights to inform obesity treatment strategies, adherence interventions, and public health communication.

## Introduction

### Background

The prevalence of obesity has more than doubled since 1990, contributing to a rise in chronic diseases associated with higher body weight, including type 2 diabetes (T2D) and cardiovascular diseases [1]. Consequently, the need for effective interventions to address obesity is urgent. Although lifestyle interventions (diet and exercise) are considered first-line strategies for weight management, they are often ineffective in the long term [2,3]. The few pharmacotherapies approved for treating overweight and obesity have historically produced only modest results, typically achieving 5%-10% weight loss [4]. Moreover, although bariatric surgery is effective for many patients, it carries significant morbidity and mortality risks [5,6]. Accordingly, the advent of glucagon-like peptide-1 receptor (GLP-1R) agonists, which are highly effective in promoting substantial weight loss and improving related health outcomes, has dramatically redefined the treatment landscape for these conditions.

Semaglutide, an incretin mimetic, is a GLP-1R agonist that stabilizes blood glucose by stimulating insulin secretion and inhibiting glucagon production [7]. It delays gastric emptying and influences appetite-regulating neural pathways, increasing satiety and reducing food intake in some individuals [8]. The first FDA-approved formulation of semaglutide was Ozempic, a once-weekly subcutaneous injection for the management of T2D [9]. This regimen offered the convenience of less frequent dosing compared with the previously approved incretin mimetic liraglutide, which required daily injections [7,10,11]. A substantial body of clinical trial data now indicates that, compared with placebo, Ozempic significantly reduces body weight (7.9%-17.3%) [12-16], lowers HbA_1c_ (glycated hemoglobin) levels, waist circumference, and systolic blood pressure, and improves overall physical functioning [13,14,16]. In clinical trials, improvements in these outcomes are generally observed within 3 months of initiating treatment [14,16-18]. In light of these findings, there has been intense interest in the off-label use of Ozempic for cosmetic weight loss, fueled in part by its popularization in mainstream and social media [19], despite other semaglutide formulations, such as Wegovy, being specifically approved for the treatment of obesity [20].

Despite their efficacy in promoting weight loss, semaglutide treatments are associated with several adverse events that vary in severity. Gastrointestinal complaints are the most common, with prevalence ranging from 41.9% to 82.8% (more common at higher doses), and include symptoms such as nausea, vomiting, constipation, and diarrhea [12-16]. Less frequent adverse events include headache, allergic reactions, and gallbladder-related disorders [15]. Adverse events are a major contributor to nonadherence to weight loss medications, including GLP-1R agonists [21-23]. In the SUSTAIN-6 clinical trial of injectable semaglutide, 22.6% of patients discontinued treatment prematurely during the 24-month study period. However, “real-world” discontinuation rates appear to be much higher. One study reported a 12-month discontinuation rate of 33% following initiation of once-weekly Ozempic therapy [24], while another study, which did not differentiate between different forms of injectable GLP-1R agonists, reported that 70.1% of patients discontinued treatment within 24 months [25].

Thus, among individuals using Ozempic for weight loss, there may be a conflict between experiencing the desired effects of treatment and managing its associated side effects. Understanding how patients navigate this trade-off is essential for optimizing adherence and maximizing therapeutic outcomes.

### Aims

Here, we employed a mixed methods infoveillance approach to examine attitudes toward Ozempic among individuals with lived experience of off-label use. Specifically, we conducted a thematic analysis of user-generated reviews posted on Drugs.com [26], followed by quantitative modeling to assess how emergent themes were associated with perceived efficacy and treatment discontinuation. A major advantage of this approach is that it is not constrained by the a priori hypotheses typical of traditional quantitative designs, allowing for the emergence of unsolicited insights that might otherwise be overlooked [27]. As a form of infodemiological research, this study illustrates how publicly available, user-generated data can serve as a powerful resource for capturing patient-centered perspectives on medication effectiveness, tolerability, and real-world barriers to adherence. Despite the inherent limitations of using self-selected, anonymous online data, these findings provide unique and timely insight into how individuals evaluate the benefits and drawbacks of off-label Ozempic use for weight loss.

## Methods

### Data Collection

Data consisted of reviews of Ozempic submitted to Drugs.com, a website that provides peer-reviewed and independent information on more than 24,000 prescription drugs, over-the-counter medicines, and natural products. A unique feature of Drugs.com is its platform that allows members of the public to submit open-ended reviews and quantitative ratings of specific medications, enabling analysis of how these outcomes are related. We extracted data exclusively from respondents who selected “weight loss” as the condition for which they were using Ozempic, despite this not being an approved indication. Notably, Drugs.com has since removed “weight loss” as an option (current options now include T2D, cardiovascular risk reduction, and chronic kidney disease), making these data particularly valuable for capturing experiences of individuals using Ozempic specifically for weight loss. Using a display name, respondents are prompted to “comment on your experience with Ozempic” and are encouraged to “describe how the medication helped (or why it didn’t work); the benefits, adverse events, dosage, ease of use” in a single textbox. No demographic data are collected. Respondents can also provide a quantitative rating of the drug on a scale from 1 (not effective) to 10 (most effective) and indicate the duration of medication use.

Data were downloaded in June 2023. No retrospective time limit on reviews was imposed; the oldest review dated from February 2023, and the most recent from June 2023. User reviews of Ozempic in which weight loss was listed as the primary indication were extracted, yielding a total of 78 reviews. As described below, a total of 60 reviews were analyzed before reaching thematic saturation.

This study employed a sequential mixed methods design, integrating qualitative and quantitative analyses of user-generated data. In the first phase, an inductive thematic analysis was conducted to identify patterns and themes within users’ open-ended narratives describing their experiences with Ozempic. In the second phase, these emergent themes were quantitatively examined in relation to users’ numerical satisfaction ratings. This design was selected to provide complementary insights, capturing the contextual richness of lived experience while enabling empirical assessment of the factors most strongly associated with user satisfaction and treatment discontinuation. This approach aligns with our previously published work [27]. We acknowledge that qualitative interpretation is inherently influenced by coder perspectives; measures taken to minimize and reflect on this potential bias are detailed in the “Thematic Data Analysis” section.

### Ethical Considerations

This study analyzed secondary, publicly available, deidentified user reviews from Drugs.com. No interaction with users occurred, and no direct or indirect identifiers were collected or stored. Data-minimization procedures were applied: only text necessary for analysis was retained, quotes were screened to exclude potentially identifying details, and results are reported in aggregate wherever possible. In accordance with institutional guidance for research using publicly accessible data, this project was not subject to human participant review.

We acknowledge that, despite deidentification and public availability, online health narratives may still pose residual privacy risks (eg, potential reidentification via unique combinations of details) and may reflect audience expectations. Our use of these data was limited to analytic purposes, with careful curation of verbatim quotations and deliberate avoidance of stigmatizing language.

### Thematic Data Analysis

**Figure 1:**
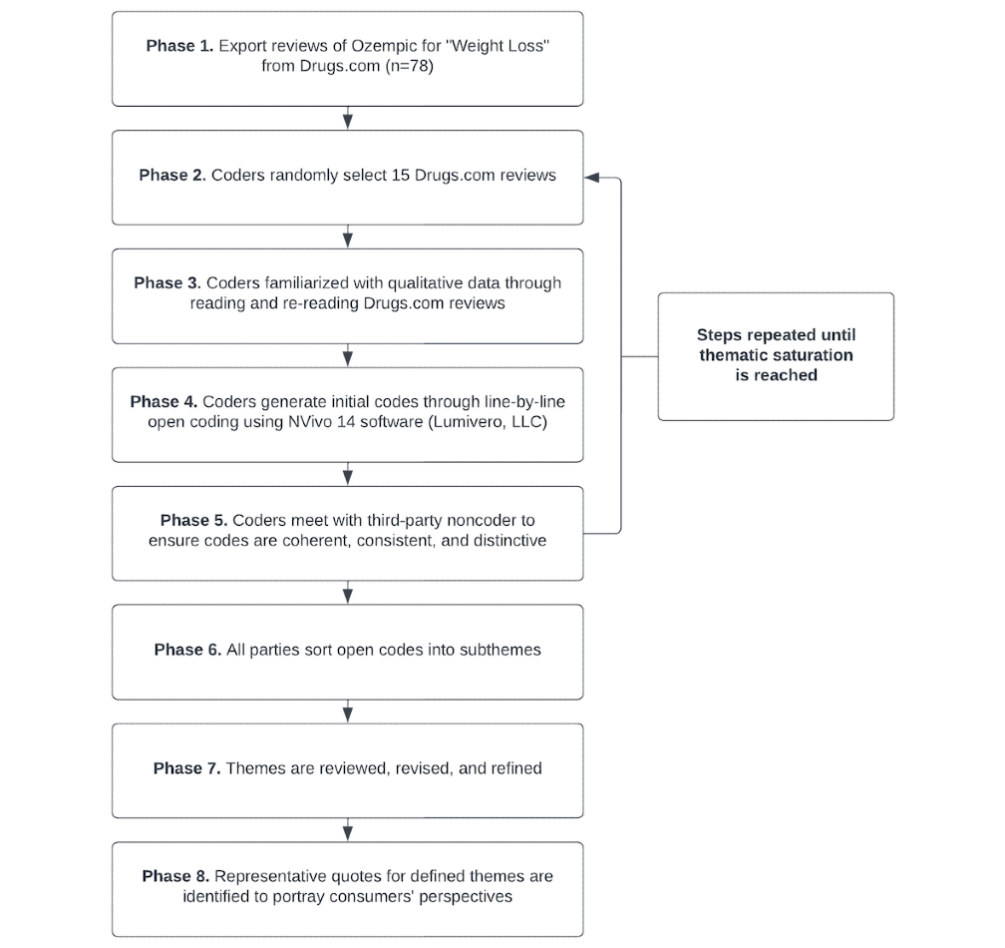
Flowchart outlining the process adopted to carry out the qualitative analysis portion of the study. First, reviews of Ozempic were extracted from Drugs.com and then randomly batched into groups of 15, with the first batch undergoing thorough familiarization via reading and rereading by the coders. Next, initial coding was conducted using NVivo 14 software, with subsequent validation by a noncoder. Similar analyses were carried out on a second batch of reviews; this process was repeated until the coders and non-coder agree that analysis of an additional batch of 15 reviews was unlikely to result in the identification of additional unique codes (i.e. thematic saturation). Codes were then organized into themes and sub-themes, which underwent iterative review and refinement. Finally, representative quotes were selected to illustrate each of the subthemes.

Qualitative data analysis was conducted using NVivo 14 software (Lumivero, LLC). Data were analyzed using a thematic analysis approach, as initially outlined by Braun and Clarke [28], following a procedure we have described previously [27] and similar to those used in studies employing alternative online data sources [29,30]. Briefly, themes were generated through an iterative process of reading through each review, suggesting themes, re-reading, and comparing categories across multiple cycles of analysis (Figure 1). To facilitate this process, the dataset was randomly divided into batches of 15 reviews. Each batch was independently reviewed by 2 coders (AJA and RMH), and excerpts relevant to the research question were coded according to a data-driven, “bottom-up” principle. This approach minimized the influence of any preconceived ideas the reviewers might have had about respondent perceptions of Ozempic. After each set of 15 reviews, both coders met with a third-party noncoder (MHJ) to compare identified codes against the original data and with each other, ensuring that the codes were coherent, consistent, and distinctive. Thematic saturation was reached when 2 consecutive batches of 15 reviews yielded no new codes or subthemes. Saturation was confirmed by consensus among the 2 coders (AJA and RMH) and the independent reviewer (MHJ), consistent with the reflexive and inductive approach to thematic analysis described previously [27,28]. The initial analysis yielded 34 distinct coding categories, which were subsequently grouped and refined into 3 overarching themes. There were no predefined criteria for determining what constituted a separate theme; rather, meaningful clusters of codes were identified, reviewed, and iteratively refined.

It is important to acknowledge that thematic coding may have been influenced by the positions and potential biases of the authors. At the time of coding, AJA (male) was a post-baccalaureate researcher (BSc with concentrations in Cell Biology and Neuroscience, Public Health, and Religion), and RMH (female) was an undergraduate student majoring in Biological Sciences on the predental medicine track. Both were conducting laboratory research on the neurobiological basis of eating disorders. MHJ (male) was a researcher with expertise in the neurobiology of motivated behaviors, including feeding. To minimize potential coder bias, the coding team underwent structured training and employed standardized procedures for codebook development. Coders met regularly to review emerging codes, reconcile discrepancies, and discuss interpretations with the independent reviewer (MHJ) throughout the analytic process.

### Quantitative Analysis of User Reviews

**Figure 2:**
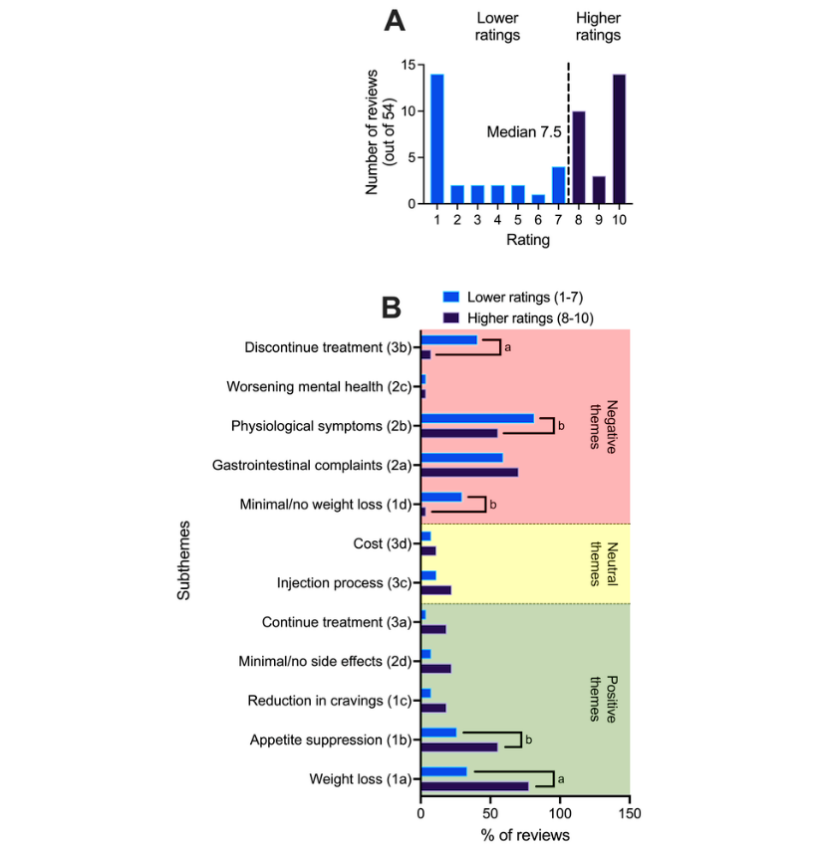
A) Histogram depicting the frequency of respondents’ quantitative ratings of Ozempic on a 1-10 scale. Data were bimodal, with the most frequent scores being 1 and 10. For subsequent analyses, we divided respondents into those who provided ratings above or below the median score of 7.5 (‘higher’ vs. ‘lower’ ratings). B) Respondents who provided higher quantitative ratings of Ozempic (8-10 out of 10) were more likely to have also contributed to subthemes associated with positive sentiment (as described by the respondents), including ‘weight loss’ and ‘appetite suppression’. Respondents who provided lower ratings (1-7 out of 10) were more likely to have contributed to several more negatively valanced subthemes, including ‘minimal/no weight loss’ ‘other physiological (nongastrointestinal) symptoms,’ and ‘plans to discontinue treatment’. Comparisons between higher vs. lower ratings made using χ^2^ analyses. *P<0.05, **P<0.01. Numbers/letters in parentheses reflect the subthemes described in the Results section.

We also sought to examine how the qualitative themes identified through thematic analysis related to the quantitative rating scores (1-10 scale) provided by respondents. Of the 60 reviews analyzed, 54 included an associated quantitative rating. We calculated the median user rating for participants whose reviews contributed to each subtheme. Based on a frequency histogram of rating scores, the data were divided into 2 groups on either side of the median score (7.5), effectively creating clusters of “higher ratings” (n=27) and “lower ratings” (n=27; see Figure 2A). A median split was used because user ratings were bimodally distributed, with most responses clustering at the extreme values (1 and 10). In this context, the median provided a more robust and meaningful threshold than the mean, enabling clearer categorical comparisons between users who expressed generally positive versus negative appraisals of Ozempic. We plotted the proportions of reviews mentioning each subtheme in the higher- and lower-rating groups, reflecting their relative frequencies within each group. For visualization purposes, each subtheme was classified according to the predominant sentiment expressed by respondents: “positive” (eg, treatment-associated weight loss), “neutral” (eg, injection process), or “negative” (eg, nausea and gastrointestinal complaints; see Figure 2B). Separate chi-square tests were conducted to compare the frequency of each subtheme’s representation between higher- and lower-rating groups. A 2-sided test with a type I error rate of 0.05 was used for all analyses.

### Reporting Standards

This study was designed and reported in accordance with the Good Reporting of a Mixed Methods Study (GRAMMS; Multimedia Appendix 1) and Standards for Reporting Qualitative Research (SRQR; Multimedia Appendix 2) guidelines to ensure transparency and reproducibility.

## Results

### Thematic Analysis

#### Overview

Our thematic analysis reached saturation after analyzing 60 responses. Three major themes emerged from these analyses (outlined in Tables 1-3). Each overarching theme comprised several related subthemes that reflected a spectrum of responses (eg, weight loss vs no weight loss). Some respondents contributed to multiple related subthemes (eg, individuals who initially lost weight with Ozempic but later regained weight were included in both “Weight loss and related outcomes” and “No/minimal weight loss or weight rebound”). Below, we describe each theme and provide representative verbatim examples. For each subtheme, we also report the median quantitative rating (1-10 scale) among all participants who contributed to that subtheme, along with the respective median absolute deviation (MAD) from the median.

#### Theme 1: Change in Body Weight/Appetite

Approximately half of respondents (33/60, 55%) indicated that they experienced weight loss at some point during Ozempic treatment (Table 1; subtheme 1a). Some users also reported improvements in weight-related outcomes, including reductions in cholesterol levels. Quantitative ratings from respondents contributing to this subtheme (median 8.5, MAD 1.5) were higher than the overall group median (median 7.5, MAD 2.5). A substantial proportion of respondents (22/60, 37%) reported that Ozempic treatment was associated with appetite suppression (Table 1; subtheme 1b). The median quantitative ratings for this group were similar to those for subtheme 1a (median 8.0, MAD 1.5), reflecting the considerable overlap in respondents (n=15) contributing to both subthemes. Additionally, a subtheme emerged regarding a reduction in food cravings (Table 1; subtheme 1c), with 8 of 60 (13%) respondents reporting that Ozempic treatment was associated with decreased cravings, particularly for sugary and greasy foods (median 10.0, MAD 0.0). A total of 40 unique respondents were coded under subthemes 1a, 1b, or 1c, indicating that 40 out of 60 (67%) respondents reported reductions in weight, appetite, or cravings. By contrast, 11 out of 60 (18%) respondents expressed no/minimal weight loss or weight rebound (Table 1; subtheme 1d; median 1.0, MAD 0.0). Among these, some noted they had not lost any weight, while others reported weight loss occurring slowly. Four respondents in subtheme 1d also appeared in subthemes 1a, 1b, or both, suggesting that although they initially experienced reductions in weight or appetite, these effects were not sustained over time (ie, weight loss plateaued or reversed).

**Table 1:** Representative quotes for theme 1: “Change in Body Weight/Appetite.”

Subtheme	n	Median (median absolute deviation) drug rating (0-10)	Examples of review comments (and drug rating associated with comment)
1a. Weight loss and related outcomes	33	8.5 (1.5)	I’ve been taking Ozempic for almost 1 year and I have lost 55 lbs. [Rating N/A^a^] For the first time in 30 years I don’t go to bed kicking myself for what I’ve eaten or making promises to myself to make amends for overeating. [Rating 7] I started at 192 lbs Nov 15th and as of May 1st I weigh 152 lbs. [Rating 10] I am more than pleased that my cholesterol is now 180 from 270 on medication. I have never been below 200 total cholesterol in my life! [Rating 10] In the first month, I lost 15 pounds on the lowest dose. [Rating 10]
1b. Appetite suppression	22	8.0 (1.5)	I don’t have much an appetite and I feel fuller faster and longer. [Rating 10] My appetite was reduced by 90%. I used to overeat, but now I can only manage two small meals a day. [Rating 9] It curbed my appetite from the moment I took the 1st dose. I didn’t feel any hunger despite being on a low-calorie diet and exercising five times a week. [Rating 10] Yes, it makes you eat way less, I was never hungry but made myself eat because I had to. [Rating 2]
1c. Reduction in food cravings	8	10.0 (0.0)	I found my sugar cravings disappeared once I started taking 1 mg. Up until then I still craved sugary foods. I lost all interest in greasy food (fries, anything deep fried etc) from .5 mg and up. [Rating 10] Ozempic helped me cure my sugar addiction and greediness. [Rating 10] I don’t crave junk food and only eat 1/3 of what I used to since I stay full longer. It’s nice [Rating 8]
1d. No/minimal weight loss or weight rebound	11	1.0 (0.0)	I am loosing [sic] weight but it is very slow (.25/week if I am lucky) [Rating 5] It has done absolutely NOTHING for me for weight loss. [Rating 1] it seems to have plateaued as I haven’t lost any weight since Christmas and it’s now March. [Rating N/A] To date, I am not losing anything and most weeks I have gained the weight back. [Rating 1]

^a^N/A: not applicable.

#### Theme 2: Nonweight-Related Symptoms and Side Effects

The majority of respondents (48/60, 80%) indicated that Ozempic treatment was associated with nonweight-related symptoms and side effects that varied in nature and severity. Nausea and gastrointestinal complaints were the most frequently reported (Table 2; subtheme 2a; 37/60, 62%), including general nausea, vomiting, burping, and severe constipation. Interestingly, the quantitative ratings associated with this subtheme (median 8.0, MAD 2.0) were similar to the overall median across all respondents (median 7.5, MAD 2.5), suggesting that the presence or absence of these symptoms was not a decisive factor in participants’ overall appraisal of the medication (discussed further below). Respondents also described a range of other physiological (nongastrointestinal) symptoms, including headaches, gallbladder complications, severe dehydration, blood loss, and anemia (Table 2; subtheme 2b; 40/60; 67%; median 7.0, MAD 3.0). Two respondents reported negative impacts on mental health, specifically the onset or worsening of depressive symptoms (Table 2; subtheme 2c; median 4.5, MAD 3.5). Finally, a minority of respondents (8/60, 13%) explicitly indicated that they did not experience any distressing adverse events (Table 2; subtheme 2d; median 10.0, MAD 0.0).

**Table 2:** Representative quotes for theme 2: “Nonweight-Related Symptoms and Side Effects.”

Subtheme	n	Median (median absolute deviation) drug rating (0-10)	Examples of review comments (associated quantitative rating of Ozempic efficacy)
2a. Nausea and gastrointestinal complaints	37	8.0 (2.0)	It started with huge belches and nausea. That night I vomited and was so lethargic and nauseous that I didn’t get out of bed for 3 days. [Rating 5] The most consistent symptom throughout my 5 months on Ozempic has been severe constipation. The inability to down lots of water like I used to has only added to the constipation. Nausea has also been prevalent from early on but reached the point of unbearable after a few weeks on 2 mg. Started throwing up daily around that time as well which was when the costs started outweighing the benefits. [Rating 7] I have had some very bad nausea, vomiting and diarrhea. Also lots of burping and it smells terrible. When I have vomited - it is sooo much volume. More than anytime in my life. [Rating 7] I have had massively bad headaches, nausea, vomiting, and stomach pain. After the first injection, I ended up in the ER because of my stomach pain, and then again 5 days later. I haven’t been able to keep anything down. I can barely keep 2 sips of water down. I can’t even take any of my prescription medications because I am constantly throwing them back up. [Rating 3]
2b. Other physiological (non-gastrointestinal) symptoms	40	7.0 (3.0)	I waited for three weeks and then tried again. However, after two injections, I became severely dehydrated and ended up in the ICU at the Heart Center. I had collapsed at a baby shower due to dehydration and was experiencing blood loss in my stools, anemia, and electrolyte imbalances. I could have died, but thankfully, I am still here to share my story. [Rating 1] I’ve gotten a few headaches [Rating 10] The sad news is I got gallbladder problems from ozempic and my gallbladder has to be removed. [Rating 1] However, after a couple of months, I started getting abdominal pains in the upper right quadrant that extended to my back, between my shoulders. The pains would manifest a day or two after the shots and last several hours. It was worse during standing or walking and not a problem when sitting. [Rating 8] I do get a case of terrible heartburn after each injection. [Rating 1] ...swelling of the throat, and difficulty swallowing which has not stopped since the first and last dose [Rating 1] I have constant dizziness [Rating 4] I had weird sores on my tongue. [Rating 5]
2c. Detrimental mental health outcomes	2	4.5 (3.5)	The worst side effect for me was depression. I would have mild anxiety and this drug made it a lot worse and made my mood very low...I just couldn’t stick feeling so low in my mood. [Rating 8] Depression - very strange feeling, almost like out of body experience. For first time found myself HATING body...Difficulty focusing. [Rating 1]
2d. Minimal or no experience with side effects	8	10.0 (0.0)	Obviously some people have terrible side effects, but I’ve had none (not even a headache!). [Rating 10] I haven’t had any side effects, only positive ones! [Rating 10] No side effects whatsoever but saying that I was (and still am) eating healthy (no greasy/fried food, no sugar, no alcohol, low fat). [Rating 10]

#### Theme 3: Plans for Ongoing Use Versus Discontinuation

Some respondents (n=20) discussed their intentions regarding continuation or discontinuation of Ozempic treatment, as well as practical considerations related to ongoing use. A small subset (n=6) explicitly reported plans to continue treatment (Table 3; subtheme 3a; median 9.0, MAD 1.0), often despite experiencing side effects. By contrast, a larger number (n=14) explicitly indicated plans to discontinue Ozempic (Table 3; subtheme 3b), and their quantitative ratings were correspondingly lower than the overall group median (median 3.0, MAD 2.0). Across the entire sample, 10 out of 60 (17%) respondents discussed the injection process associated with Ozempic administration (Table 3; subtheme 3c; median 8.0, MAD 1.0). Most users described the process as straightforward and convenient, with only 3 reporting difficulties. A small number (n=5) mentioned the cost of Ozempic (Table 3; subtheme 3d; median 8.0, MAD 1.0); while some considered it manageable when covered by insurance or medical cards, others identified cost as a barrier to continued use.

**Table 3:** Representative quotes for theme 3: “Plans for Ongoing Use Versus Discontinuation.”

Subtheme	n	Median (median absolute deviation) drug rating (0-10)	Examples of review comments (associated quantitative rating of Ozempic efficacy)
3a. Plans to continue with treatment	6	9.0 (1.0)	I have had all 5 of the main side effects, nausea, stomach pain, vomiting, diarrhea, and constipation. I am happy with the weight loss, so am learning to manage these. [Rating 8] The side effects at the beginning were worth it for me but from the sounds of it, mine weren’t that bad. I have never once thrown up. [Rating 10] What it has done is to force me to give up bad habits because I do not like staying nauseous. I do believe this negative reinforcement will make me sustain weight loss after I have finished my rounds. [Rating 10]
3b. Plans to discontinue treatment	14	3.0 (2.0)	I do not plan to continue. No pain, no gain. I’ll get my loss the old-fashioned way with proper diet and exercise. Never again want to feel this way on Ozempic. [Rating 1] ...while watching TV, I heard an Ozempic commercial that included warnings about gallbladder problems and pancreatitis. I immediately stopped using it and went to my doctor. [Rating 1] I have had pretty much all the side effects possible. I have missed four days of work because I can’t leave my bathroom. I can’t keep anything in my stomach with it hurting and the diarrhea will not stop. I’m getting dehydrated but If I drink it goes right through me. I have a family to look after and I can’t. All I want to do is sleep. I don’t think I will be taking my second injection. [Rating N/A^a^] I've been on Ozempic for 4 months. Recently raised my dose to .75 mg but I am stopping this medication. Yes, I lost 15 lbs but suffered with the worse sulfur gas, was sick in bed at times, missing appointments because I felt that crap. [Rating 2] This is my third week and I feel absolutely horrid!! I have constant dizziness, extreme fatigue, and generally feel like crap. I don't think I will continue. It's not worth it to lose maybe 20 lbs but can't get out of bed and function. I am tired of feeling like death warmed over. [Rating 4] Got up to 2mg after 2 years at 1mg. Stopped because I began vomiting and feeling nauseous [Rating 7]
3c. Injection process	10	8.0 (1.0)	I had to go to my doctor’s office to have them me show how to use the pen from priming it to injecting the pen. It was easy. Even the directions from the website is easier. [Rating 10] It is very convenient to use (inject once a week), and the needle is so thin (less than a hair) you don’t even feel it. [Rating 10] Dosing 2mg is difficult as a needle change is required. [Rating 7] Incorrect filling of the pen, it never has enough according to dosage. [Rating N/A]
3d. Cost	5	8.0 (1.0)	I’m in Ireland, and it costs €150 a month for four injections, which are covered by my medical card. [Rating 9] Unfortunately, it did take a toll on my wallet, and I eventually had to switch from my hospital/GP to getting it online by telehealth from semalean. My son has also started taking it, and I would recommend it to anyone who is curious, but only if they are willing to see if the side effects apply to them. If they do not, and the treatment is affordable or covered by insurance, it can be truly amazing. [Rating 8] Don’t waste your money on this stuff. [Rating 1]

^a^N/A: not applicable.

### Identification of Themes Contributing to Higher Versus Lower Quantitative Ratings of Ozempic Efficacy

Across the 54 participants who provided a quantitative rating of Ozempic’s efficacy on the 1-10 scale, the average rating was 5.98 (SD 3.65), and the median was 7.5 (MAD 2.5; Figure 2A). For comparison, the average rating among all available user reviews of Ozempic for weight loss on Drugs.com at the time of data collection (n=78) was 6.04, with a median of 7.0 (MAD 3.0). Statistical analysis indicated no significant difference between these 2 distributions (Mann-Whitney *U* test, *P*=.996), indicating that our analytic subsample was representative of the broader dataset. The most frequent scores were 1 and 10 (n=14 each), indicating that more than half of respondents (28/54, 52%) rated Ozempic as either the highest or lowest possible score (see Figure 2A). Based on the distribution of user ratings, we divided the data into 2 groups using a median split: those with higher ratings (scores of 8-10; n=27) and those with lower ratings (scores of 1-7; n=27; Figure 2A). To examine which qualitative subthemes were associated with more positive versus negative evaluations of Ozempic, we conducted 2 × 2 chi-square tests comparing the frequency of each subtheme across the higher- and lower-rating groups (Figure 2B).

As expected, several subthemes reflecting positive sentiment were more frequently observed among respondents who provided higher ratings of perceived efficacy than among those who provided lower ratings. These included weight loss (subtheme 1a; 21/27, 78%, vs 9/27, 33%; *χ*^2^_1_[n=54]=10.80, *P*=.001) and appetite suppression (subtheme 1b; 15/27, 56%, vs 7/27, 26%; *χ*^2^_1_[n=54]=4.91, *P*=.03). Although reduction in food cravings was mentioned more often among higher-rating respondents (subtheme 1c; 5/27, 19%, vs 2/27, 7%), this difference did not reach statistical significance (*P*=.22). Three subthemes reflecting negative sentiment were observed at significantly higher frequencies among respondents providing lower ratings. These included no/minimal weight loss or weight rebound (subtheme 1d; 8/27, 30%, vs 1/27, 4%; *χ*^2^_1_[n=54]=6.53, *P*=.01), physiological (non-gastrointestinal) symptoms (subtheme 2b; 22/27, 81%, vs 15/27, 56%; *χ*^2^_1_[n=54]=4.21, *P*=.04), and plans to discontinue treatment (subtheme 3b; 11/27, 41%, vs 2/27, 7%; *χ*^2^_1_[n=54]=8.21, *P*=.004). Interestingly, the frequency of nausea/gastrointestinal complaints (subtheme 2a; 19/27, 70%, vs 16/27, 59%; *χ*^2^_1_[n=54]=0.73, *P*=.39) did not differ significantly between the higher- and lower-ratings groups.

**Figure 3:**
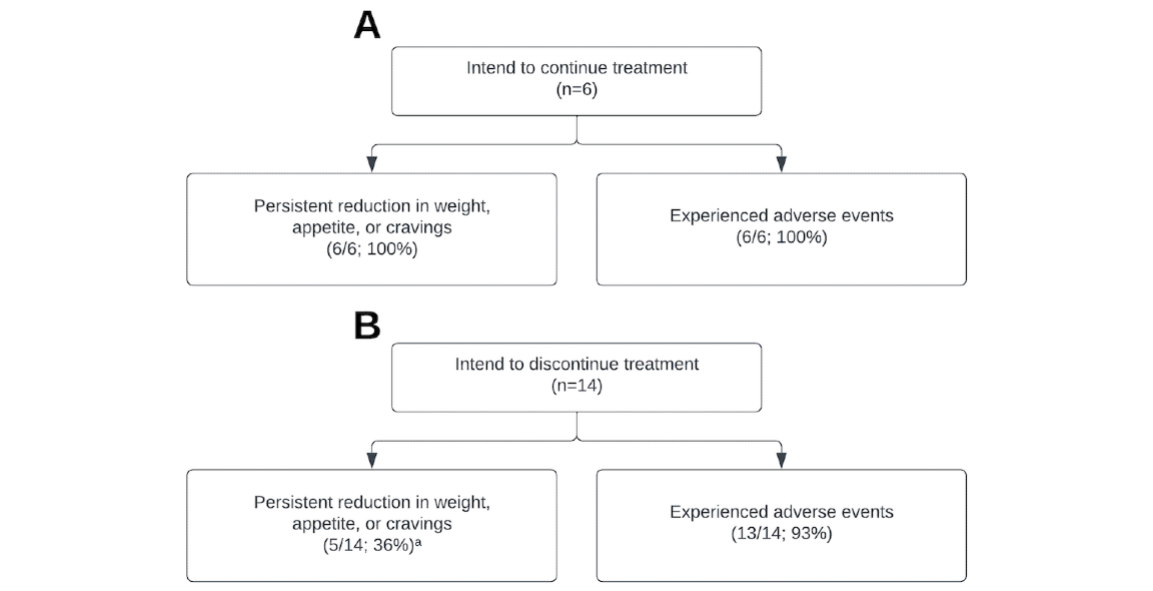
Weight loss and side effect outcomes among respondents who explicitly indicated an intention to continue (n=6) or discontinue (n=14) Ozempic treatment in the long term. Among those intending to continue (A), 100% reported ongoing weight loss, appetite suppression, and/or reduction in cravings (i.e., subthemes 1a, b, c, but not d), as well as experiencing some type of side effect (subtheme 2a, b, and/or c). Users who intended to discontinue treatment (B) were significantly less likely to report ongoing weight loss and/or appetite suppression (36%) and had a high frequency of adverse events (93%). **p<0.01, χ2 analyses).

Finally, we conducted exploratory analyses to better understand the profile of the relatively small number of respondents who explicitly indicated their intention to continue (n=6; Figure 3A) versus discontinue (n=14; Figure 3B) Ozempic treatment. All respondents (6/6, 100%) who intended to continue treatment reported ongoing weight loss, appetite suppression, or reduction in food cravings (ie, subthemes 1a, 1b, and 1c, but not 1d); this proportion differed significantly from those who intended to discontinue treatment (5/14, 36%; *χ*^2^_1_[n=20]=7.01, *P*=.008). By contrast, both groups reported a high frequency of adverse events (subthemes 2a, 2b, or 2c; 6/6, 100%, vs 13/14, 93%; *P*=.50). All 6 continuers reported nausea and gastrointestinal complaints (subtheme 2a), and 4 experienced other physiological symptoms (subtheme 2b). Similarly, 9 of the 14 discontinuers experienced nausea, and 12 reported other physiological symptoms. Together, these data suggest that the intention to discontinue Ozempic may be driven primarily by a lack of perceived efficacy (failure to lose weight), rather than by side effects or adverse events.

## Discussion

### Principal Findings and Comparison to Prior Work

This study provides new insight into how individuals perceive and evaluate the off-label use of Ozempic for weight loss, based on unsolicited, real-world data from an online medication review platform. Using a mixed methods approach, we found that user satisfaction was driven primarily by perceived effectiveness in promoting weight loss and appetite suppression, whereas gastrointestinal side effects were common but exerted limited influence on overall evaluations or decisions to continue treatment. Rather, discontinuation was most strongly associated with no/minimal weight loss or the occurrence of other, nongastrointestinal side effects. These findings highlight that, for many users, perceived efficacy outweighed tolerability concerns—a perspective that may be underrepresented in traditional clinical trials—and demonstrate the potential of infoveillance methods to capture patient-centered attitudes that shape treatment adherence.

Of the 60 respondents, 40 (67%) reported reduced weight, appetite, or cravings as a result of Ozempic treatment. This finding aligns with clinical trial data demonstrating the broad efficacy of semaglutide in promoting weight loss, with reductions of up to 17.3% observed after approximately 1 year of treatment, depending on dose and patient population [12,14,15]. Across these studies, approximately 13.5% of participants failed to achieve ≥5% weight loss with semaglutide 2.4 mg, comparable to the 18% (11/60) of participants in our sample who reported minimal or no overall weight loss. Consistent with these findings, subthemes related to weight outcomes were major contributors to respondents’ overall quantitative ratings of Ozempic: subthemes 1a and 1b (“weight loss and related outcomes” and “appetite suppression”) were associated with higher overall ratings, whereas subtheme 1d (no/minimal weight loss or weight rebound) occurred more frequently among lower ratings. Moreover, reductions in weight, appetite, and cravings (subthemes 1a, 1b, and 1c) were strongly associated with respondents’ intention to continue versus discontinue treatment, underscoring these outcomes as being closely associated with long-term medication adherence. Notably, a retrospective study of electronic health records in the United States reported that semaglutide treatment was associated with higher persistence rates at 1 year (40%) compared with other weight loss medications, including liraglutide (17%), phentermine-topiramate (13%), and naltrexone-bupropion (10%) [31], likely reflecting its superior efficacy in promoting weight loss.

Several respondents indicated that although Ozempic treatment initially led to weight loss, this effect had plateaued or even reversed with continued use. This observation aligns with evidence from clinical studies showing that weight loss tends to plateau after approximately 1 year of semaglutide treatment, with weight regain often emerging during the second year in trial extension cohorts [32]. Such plateaus are consistent with those observed following other weight loss interventions and are thought to reflect metabolic adaptations, including reductions in resting and nonresting energy expenditure, accompanied by compensatory changes in appetite-regulating hormones [2,33-35]. In our dataset, very few respondents reported their treatment duration; therefore, we were unable to determine whether this variable mediated positive versus negative appraisals of Ozempic’s efficacy. This should be a focus of future research, as it is conceivable that patient attitudes toward Ozempic become increasingly negative as weight loss plateaus or reverses over the longer term. Such dynamics likely have important implications for long-term medication adherence, including for semaglutide formulations specifically approved for obesity and overweight [36].

A high proportion of respondents reported experiencing gastrointestinal complaints, including nausea, diarrhea, and vomiting. This aligns with clinical trial data identifying these as the most common adverse events associated with semaglutide treatment, with prevalence ranging from 41.9% to 82.8% across studies [12-16], as well as with preclinical evidence that GLP-1R agonists act on hindbrain regions involved in emesis control [37]. In our sample, these adverse events occurred with similar frequency among respondents who provided higher versus lower quantitative ratings, suggesting that gastrointestinal symptoms did not substantially influence overall attitudes toward Ozempic as a weight loss medication. This aligns with data from a previous study showing that 99.5% of gastrointestinal adverse events were nonserious, transient, and occurred most frequently during or shortly after dose escalation [38]. Moreover, across several clinical trials, treatment discontinuation due to gastrointestinal complaints was relatively uncommon, affecting only 3.4%-4.2% of participants [14,16]. Together, these findings indicate that gastrointestinal side effects are generally well tolerated and often regarded as “acceptable,” particularly among individuals who experience meaningful weight loss (as described by 1 respondent: “I have had all 5 of the main side effects, nausea, stomach pain, vomiting, diarrhea, and constipation. I am happy with the weight loss, so am learning to manage these”). By contrast, users who reported other physiological (nongastrointestinal) symptoms tended to give lower quantitative ratings. This may reflect the greater severity of some of these adverse events, with several users indicating hospitalization due to complications such as severe dehydration or gallbladder removal. Although we cannot confirm that these outcomes were directly attributable to Ozempic treatment, serious treatment-associated adverse events have been reported in approximately 10% of participants in large-scale studies [15,16], including gallbladder disorders such as cholelithiasis and cholecystitis, which have led to treatment discontinuation in some cases [12].

Our data also included 2 instances in which respondents reported experiencing depression symptoms that they attributed to Ozempic treatment. Recent discussions have raised concerns about a possible association between semaglutide use and adverse mental health outcomes, particularly suicidal ideation [39]. This aligns with a recent FDA submission noting a disproportionate number of reports of “depression/suicidal” and suicidal ideation among individuals treated with semaglutide, although no causal relationship was established [39]. However, other studies, including a recent meta-analysis of 25 clinical trials, have found no association between GLP-1R agonists and suicidal or self-injurious behaviors [40,41], and some evidence even suggests a lower risk of these outcomes compared with other medications for obesity and T2D [42]. These mixed findings mirror patterns observed among bariatric surgery patients, where treatment has been associated not only with improvements in depression and anxiety but also with an increased risk of suicidality and self-injurious behavior [43]. Collectively, these data highlight the need for further research, including prospective studies and controlled clinical trials, to clarify the potential mental health risks associated with semaglutide use. Complementary preclinical investigations may also be necessary to identify shared neurobiological pathways underlying the regulation of appetite and mood.

### Limitations and Future Directions

We acknowledge several important limitations of our study. First, our data were opportunistic and derived exclusively from a single website (Drugs.com), which does not publish demographic information about its users. As such, it is unclear to what extent our findings are representative of the broader population of Ozempic users. Online reviewers may also differ systematically from the general treatment population (eg, in health literacy, socioeconomic status, or engagement with digital health platforms), potentially biasing the types of experiences shared. Furthermore, self-selection biases may amplify extreme positive or negative perspectives, leading to an overrepresentation of polarized views [44]. Although we mitigated this by including all eligible reviews and reporting aggregate rather than individual data, future research should extend these findings through prospective, consented studies that collect demographic and clinical information, enabling the hypotheses generated here to be tested in more representative and generalizable samples.

Second, cumulative evidence indicates that weight loss in response to GLP-1R agonists may be more pronounced among women [45-47]; future research should therefore examine whether perceptions of Ozempic’s clinical benefits and side effect profile differ by sex. Relatedly, because the data were self-reported, we could not independently verify clinical outcomes, adverse events, or the reasons for Ozempic use. Although this limitation may introduce some inaccuracy, the strong consistency of themes across respondents provides reassurance regarding the reliability of the data. Third, weight loss outcomes and adverse events are likely influenced by both the dose of Ozempic and the duration of treatment. These variables were not available in the present dataset and therefore, could not be analyzed. Finally, online reviews of consumer products, including medications, may be shaped by contextual factors such as prior reviews or platform norms, which could “prime” respondents to emphasize certain outcomes over others. This limitation underscores the need to triangulate infoveillance data with controlled, prospective designs to validate and extend these findings.

### Clinical Implications

These findings offer practical insights for both clinicians and their patients. For clinicians, acknowledging that side effects are common, and occasionally serious, can help guide expectation-setting, safety monitoring, and decisions about when to consider alternative treatments. For patients, understanding that side effects vary in severity and that weight loss may plateau over time can support more realistic expectations and informed discussions with health care providers about whether to continue or adjust therapy. Together, these insights can foster clearer communication and more patient-centered decision-making.

### Ethical Implications of Using Deidentified Online Data

Although we analyzed public, deidentified posts, we acknowledge that ethical considerations remain, including (1) the potential for reidentification through rare combinations of clinical details; (2) users’ possible expectation that posts were intended for peer support rather than research; and (3) the risk of unintended harm, such as reinforcing stigma around the use of medications for weight loss. We mitigated these risks by limiting data collection to information necessary for analysis, screening quotations to remove potentially identifying details, reporting results in aggregate, and using neutral, nonsensational language. Future research should build on the insights from this study through prospective designs with informed consent, enabling hypotheses generated here to be tested in more representative and ethically robust samples that include limited demographic and clinical data.

### Conclusion

Attitudes toward Ozempic were shaped primarily by its perceived effectiveness in promoting weight loss, with gastrointestinal side effects exerting minimal influence on overall satisfaction. For many users, the benefits of appetite suppression and weight reduction outweighed treatment-related discomfort. By leveraging an infoveillance approach, this study identified key patient-reported factors driving satisfaction and discontinuation that may be underrepresented in traditional research. These findings provide a foundation for future structured studies aimed at improving adherence and optimizing treatment strategies for individuals with overweight and obesity.

## Appendix

Multimedia Appendix 1 GRAMMS (Good Reporting of a Mixed Methods Study) checklist.

Multimedia Appendix 2 SRQR (Standards for Reporting Qualitative Research) checklist.

## Abbreviations

GLP-1R: glucagon-like peptide-1 receptor
GRAMMS: Good Reporting of a Mixed Methods Study
HbA1c: glycated hemoglobin
MAD: median absolute deviation (from median)
SRQR: Standards for Reporting Qualitative Research
T2D: type 2 diabetes

## Data Availability

The data that support this study are available upon reasonable request from the corresponding author.
