# Supplementary material for: Patient Perceptions of Ozempic (Semaglutide) for Weight Loss: Mixed Methods Analysis of Online Medication Reviews
Source: J Med Internet Res. 2026 Jan 9;28:e78391. doi: 10.2196/78391 (PMC12904099; doi:10.2196/78391)
Supplement: Multimedia Appendix 1 [file jmir_v28i1e78391_app1.pdf]

Good Reporting of a Mixed Methods Study (GRAMMS) Checklist

Guideline Section	Page(s)	Description
Describe the justification for using a mixed-methods approach to the research question	Design: p7–8	The study combined thematic (qualitative) and quantitative analyses to integrate depth of lived experience with measurable user ratings, providing a comprehensive understanding of perceptions of Ozempic use for weight loss.
Describe the design in terms of the purpose, priority, and sequence of methods	Design: p7–8	A sequential explanatory design was used: qualitative analysis of user reviews was conducted first to identify emergent themes, followed by quantitative analyses examining associations between these themes and satisfaction ratings.
Describe each method in terms of sampling, data collection, and analysis	Data collection: p8–9; Data analysis: p9–11	User reviews were selected from Drugs.com under the "weight loss" indication. Reviews were read, coded, and analyzed inductively in NVivo 14 by two independent coders until thematic saturation. Quantitative data (user-provided ratings) were analyzed using descriptive statistics and chi-square tests.
Describe where integration has occurred, how it has occurred, and who has participated in it	Design: p8–9	Integration occurred during the quantitative analysis and interpretation phases. Qualitative subthemes were linked to numerical ratings and discussed collaboratively among the coding team and senior author to interpret convergence and divergence between methods.
Describe any limitation of one method associated with the presence of the other method	Strengths and limitations: p21–22	Qualitative analysis was limited by the absence of demographic and dosing data that would have strengthened quantitative modeling. Conversely, quantitative findings could not capture the full nuance of user experiences.
Describe any insights gained from mixing or integrating methods	Discussion: p17–20	Integrating qualitative and quantitative data revealed that satisfaction with Ozempic was primarily driven by perceived weight-loss efficacy rather than side-effect burden—an insight that would not have emerged from either method alone.

Adapted from: O'Cathain A, Murphy E, Nicholl J. The quality of mixed methods studies in health services research. J Health Serv Res Policy. 2008;13(2):92–98. doi:10.1258/jhsrp.2007.007074
