# Supplementary material for: Patient Perceptions of Ozempic (Semaglutide) for Weight Loss: Mixed Methods Analysis of Online Medication Reviews
Source: J Med Internet Res. 2026 Jan 9;28:e78391. doi: 10.2196/78391 (PMC12904099; doi:10.2196/78391)
Supplement: Multimedia Appendix 2 [file jmir_v28i1e78391_app2.pdf]

Standards for Reporting Qualitative Research (SRQR) Checklist

Section / Item	Where Reported in Manuscript	Description
Title and abstract - Title	Title page	Title identifies the study as a “mixed methods analysis” of patient perceptions of Ozempic.
Title and abstract - Abstract	Abstract	Structured abstract includes rationale, objectives, qualitative and quantitative methods, and main findings.
Problem formulation	Background (p.3–5)	Provides context on obesity, GLP-1 receptor agonists, and gaps in patient-reported outcome data regarding off-label Ozempic use.
Purpose or research question	Aims (p.5)	States aim to examine user-reported experiences of off-label Ozempic for weight loss and identify factors associated with satisfaction and discontinuation.
Qualitative approach and research paradigm	Thematic Data Analysis (p.9)	Reflexive thematic analysis following Braun & Clarke (2006); inductive, data-driven approach emphasizing constructivist interpretation.
Researcher characteristics and reflexivity	Thematic Data Analysis (p.10)	Describes coder demographics, training, and expertise in eating-disorder neurobiology; acknowledges influence of researcher perspectives and use of consensus discussions to reduce bias.
Context	Data Collection (p.8)	Data derived from a public online medication-review platform (Drugs.com), reflecting real-world, self-reported experiences.
Sampling strategy	Data Collection (p.8–9)	Reviews selected if “weight loss” listed as indication; thematic saturation reached after 60 reviews.

Ethical issues pertaining to human participants	Ethical Considerations (p.8)	Analysis of publicly available, anonymous data deemed exempt from human-subjects review; privacy safeguards and data minimization applied.
Data collection methods	Data Collection (p.8–9)	Reviews manually extracted (February–June 2023), entered into NVivo 14, and analyzed iteratively by two coders in batches of 15.
Data collection instruments and technologies	Data Collection (p.8–9)	NVivo 14 software used for coding and theme development; Drugs.com provided structured quantitative fields (ratings, comments).
Units of study	Data Collection (p.8–9)	Sixty unique user reviews met inclusion criteria; each represents a single de-identified patient experience.
Data processing	Data Analysis (p.9–10)	Reviews imported into NVivo; usernames removed; coding logs and memos maintained to preserve analytic transparency.
Data analysis	Thematic Data Analysis (p.9–11)	Inductive thematic analysis using Braun & Clarke's six-phase process; coding conducted independently by two researchers and reviewed by a third until consensus achieved.
Techniques to enhance trustworthiness	Thematic Data Analysis (p.10)	Used multiple coders, codebook alignment, inter-coder comparison, and third-party validation to enhance analytic reliability.
Synthesis and interpretation	Results (p.11–17)	Identified three overarching themes: (1) body weight and appetite changes, (2) non-weight-related side effects, and (3) plans for ongoing use or discontinuation.

Links to empirical data	Results (p.12–16)	Representative verbatim quotations included in Tables 1–3 to illustrate each subtheme.
Integration with prior work, implications, transferability, and contributions	Discussion (p.17–20)	Findings integrated with prior GLP-1 literature; discussion highlights novel insights into user-driven risk–benefit trade-offs and implications for adherence.
Limitations	Limitations and Future Directions (p.21–22)	Addresses non-representativeness, self-selection bias, lack of demographic/dosing data, and challenges of analyzing online self-report data.
Conflicts of interest	Competing Interests (p.2)	Discloses relevant intellectual property (MHJ patent) and confirms no other conflicts.
Funding	Acknowledgments / Funding (p.23)	Lists institutional, NIH, and foundation grants supporting the study; funders had no role in design or interpretation.

Adapted from: O'Brien BC, Harris IB, Beckman TJ, Reed DA, Cook DA. Standards for reporting qualitative research: a synthesis of recommendations. Acad Med. 2014;89(9):1245–1251. doi:10.1097/ACM.0000000000000388
